# The implementation of teledentistry training in orthodontic practice: an explanatory sequential mixed-methods study

**DOI:** 10.1186/s12909-024-06509-5

**Published:** 2024-12-30

**Authors:** Supakit Peanchitlertkajorn, Charn Ngamdachakij, Boonsita Wongwatjana, Wichayaporn Jongpatranichpunth, Kawin Sipiyaruk

**Affiliations:** 1 Department of Orthodontics, Faculty of Dentistry, Mahidol University, Bangkok, 10400 Thailand; 2 Mahidol International Dental School, Faculty of Dentistry, Mahidol University, Bangkok, 10400 Thailand

**Keywords:** Dental education, Orthodontics, Outcome-based education, Teledentistry, Telehealth

## Abstract

**Background:**

Teledentistry has been increasingly used in orthodontic practice. Comprehensive and appropriate trainings should be required to enhance the effective use of teledentistry. However, there is still a lack of adequate teledentistry training in postgraduate orthodontic programs. Therefore, this research aimed to investigate the perceptions of key stakeholders regarding the necessity of teledentistry and to formulate a conceptual framework highlighting the implementation of teledentistry training in orthodontic education.

**Methods:**

This research employed an explanatory sequential mixed-method design. The research participants included orthodontic instructors, practitioners, and residents from the Faculty of Dentistry, Mahidol University. The participants were asked to complete an online questionnaire to provide initial overview of this topic. They were then purposively selected and recruited for a semi-structured interview, allowing the exploration of in-depth information.

**Results:**

Participants expressed positive perceptions toward the use of teledentistry in orthodontic practice. The conceptual framework derived from this study highlighted awareness of both advantages and concerns of teledentistry among participants, leading to the necessity of training in orthodontic education. The findings also provided in-depth information regarding expected learning outcomes, a combination of training delivery and assessment strategies to adequately prepare learners for the use of teledentistry in orthodontic practice.

**Conclusion:**

This study supports the implementation of teledentistry into orthodontic practice, with participants recognizing both its benefits and concerns. Emphasizing the significance of incorporating teledentistry preparation into orthodontic curricula, it is essential to outline expected learning outcomes, delivery methods, and assessment strategies for effective training.

## Introduction

The COVID-19 outbreak contributed to various challenges in orthodontic practice. Recent evidence confirmed its significant impact on dental practice throughout the outbreak [1]. Orthodontic care was also affected, as unexpected tooth movement could occur due to a prolonged period between visits [2]. Without proper monitoring, severe complications may arise, affecting supporting periodontal structures and teeth, including root resorption, tooth mobility, and cortical bone perforation [3]. Some minor orthodontic emergencies (e.g., bracket debonding, soft tissue irritation, and exaggerated pain) can potentially be managed or relieved through virtual consultations until an in-office visit can be scheduled [4]. Therefore, teledentistry emerges as a valuable tool for remote patient management in orthodontic practice, especially in situations when arranging in-person dental appointments poses challenges.

Even though the COVID-19 pandemic has ended, teledentistry remains a valuable tool in orthodontic practice. It facilitates collaboration among healthcare providers from different specialties to propose tailored treatments for each patient [5]. Teledentistry also has significance in fostering communication among healthcare professionals, especially in consultations between orthodontists and other dental specialists facilitating the planning of complex cases [6]. Furthermore, virtual consultations eliminate the need for patient travel, leading to potential reductions in both direct and indirect costs [7]. Given these advantages, teledentistry should be considered as a permanent armamentarium in orthodontic practice, even though the impact of the COVID-19 outbreak on dental appointments gradually diminishes.

Despite the evident benefits of teledentistry, its implementation faces several limitations and challenges. A lack of computer literacy and familiarity with available software may act as a barrier to the widespread adoption of teledentistry [8]. Concerns about the complexity of technology may lead dental practitioners to resist incorporating teledentistry into their clinical practice [6]. The associated expenses required for setting up teledentistry may serve as a disincentive to its broad acceptance [9]. Furthermore, regulations concerning teledentistry are not clearly defined in several countries [10]. There are also apprehensions about payment methods for teledentistry services [9]. While recognizing the advantages of teledentistry, it is necessary to emphasize these challenges to ensure its appropriate use with standards comparable to onsite dental visits.

To enhance the effective use of teledentistry, comprehensive and appropriate trainings should be mandatory prior to its implementation in clinical practice [11]. However, dental professionals appear to possess inadequate appreciation and knowledge of teledentistry [12]. Additionally, existing evidence indicates a deficiency in teledentistry training in dental practice [13], with no exception for postgraduate orthodontic programs. The integration of teledentistry in orthodontic practice, known as teleorthodontics, requires a deeper understanding and incorporation into training programs. Consequently, this research was conducted to investigate the perceptions of key stakeholders regarding the necessity of teleorthodontics and to develop a conceptual framework for implementing teledentistry training into orthodontic education. This research would offer valuable insights into essential topics, delivery techniques, and assessment methods crucial for establishing effective training in teledentistry for orthodontics. To achieve this, research objectives were constructed as follows:

1. To explore perceived benefits and concerns associated with the use of teledentistry in orthodontic practice.
2. To identify the teledentistry-related topics required for training in orthodontic practice.
3. To gather in-depth information on current teledentistry practices of relevant stakeholders and their suggestions for training in orthodontic practice.
4. To develop a conceptual framework for designing teledentistry training in orthodontic education, based on constructive alignment, including expected learning outcomes, and aligned teaching and assessment strategies.

## Methods

### Research design

This research employed an explanatory sequential mixed-method design, initiating with the quantitative phase conducted through an online questionnaire. Subsequently, the qualitative phase was performed to enhance the understanding of the quantitative findings. This approach offers the advantages of simplicity and the capability to delve deeper into quantitative data. The quantitative phase involved a questionnaire survey to initially capture the perceptions of key stakeholders regarding teleorthodontics. Afterwards, the qualitative phase was implemented to elucidate the quantitative data through the collection of in-depth information from semi-structured interviews. The data collection process took place between November 2021 and September 2022.

### Research participants

The participants in this research were key stakeholders involved in the implementation of teledentistry training in orthodontics, including orthodontic instructors, residents, and practitioners at the Faculty of Dentistry, Mahidol University. This diversity was intentionally sought to reflect a complete range of perceptions and experiences related to teleorthodontics. The exclusion criteria involved individuals who were no longer engaged in orthodontic practice or lacked proficiency in the Thai language. Participants for the quantitative phase were recruited through convenience sampling, with acknowledgement of the potential for non-response bias [14]. However, the impact was considered not significant, as this stage primarily served as an initial survey to generate information for the purposeful sampling and topic guide of the qualitative phase. To achieve a confidence level of 95% and a margin of error of 4%, 66 respondents were expected to complete and return the questionnaire. Following the quantitative data analysis, participants were selected using purposeful sampling for semi-structured interviews, considering factors such as age, experience, and perceptions of teledentistry in orthodontics. The sample size for semi-structured interviews was determined by data saturation, defined as the point at which no new themes emerged from additional interviews.

### Data collection tools

The questionnaire was constructed based on previous literature regarding teledentistry [7, 15, 16]. The questionnaire was structured into three sections, which were general information, perceptions related to the use of teleorthodontics, and suggestions toward teledentistry training in postgraduate orthodontic curriculum. The questionnaire was designed using a checklist format and a 5-point scale, where 1 indicated strong disagreement and 5 denoted strong agreement.

Online semi-structured interviews with a topic guide were conducted to gather in-depth information from participants. These interviews took place via a video teleconferencing platform, enabling researchers to observe participants' facial expressions and gestures. The topic guide was initially constructed based on the findings from previous literature [7, 15, 16], which was subsequently adapted following the analysis of quantitative data. The online interview was conducted in a private room to ensure the confidentiality and privacy of participants, where each session took approximately 30–45 min. Throughout the interviews, all responses provided by participants were recorded with their permission using a digital audio recorder and transcribed using a verbatim technique.

### Data analysis

Quantitative data obtained from the questionnaire were analyzed using the Statistical Product and Service Solutions (SPSS, version 28, IBM Corp.). Descriptive statistics were performed to provide an overview of data. According to the qualitative data, a framework analysis was conducted using NVivo (version 14, QSR International). This approach facilitated transparent data analysis, enabling the traceability of information back to previous stages in the research process [17]. The transparency allowed two researchers to independently analyze the qualitative data, where the emerging themes and subthemes could be discussed to obtain the consensus among all researchers, to enhance the research validity. This approach ensured that the diversity of perspectives was acknowledged and integrated in the qualitative data analysis. Subsequently, the qualitative findings were then interpreted to identify their relationships and to construct a conceptual framework.

### Ethical considerations

Ethical approval for the study was obtained by the Institutional Review Board of Faculty of Dentistry and Faculty of Pharmacy, Mahidol University on 20 October 2021 (COA.No.MU-DT/PY-IRB 2021/093.2010). All methods were performed in accordance with the relevant guidelines and regulations. Informed consent was obtained from all participants. Although the data were not anonymous in nature as they contained identifiable data, they were coded prior to the analysis to maintain confidentiality of participants.

## Results

### Research participants

There were 66 participants who completed and returned the questionnaire, consisting of 15 instructors, 15 practitioners, and 36 residents who were providing orthodontic service at the Faculty of Dentistry, Mahidol University. Following the quantitative data analysis, ten participants (three instructors, three practitioners, and four residents) were selected for semi-structured interviews. The ages of participants were presented in ranges of 5-year intervals to ensure and maintain their anonymity. Their characteristics are detailed in Table 1.

**Table 1:** Participants who participated in semi-structured interviews

Participants	Sex	Age	Experience with teleorthodontics	Self-perceived benefits	Self-perceived concerns
Instructor 1	Female	31–35	High level	High level	Low level
Instructor 2	Male	41–45	Moderate level	High level	Moderate level
Instructor 3	Male	46–50	High level	Moderate level	Moderate level
Practitioner 1	Female	51–55	Low level	High level	Moderate level
Practitioner 2	Female	61–65	Moderate level	High level	Low level
Practitioner 3	Female	46–50	High level	High level	Moderate level
Resident 1	Female	25–30	Moderate level	High level	Low level
Resident 2	Male	31–35	High level	Moderate level	Low level
Resident 3	Female	25–30	Moderate level	High level	Moderate level
Resident 4	Female	25–30	Moderate level	High level	High level

**Note:** Low level: scores ranging from 1.00 to 2.49 out of 5; Moderate level: scores ranging from 2.50 to 3.50 out of 5; High level: scores ranging from 3.51 to 5.00 out of 5

### Self-perceptions in the use of teledentistry

#### Self-perceived benefits in the use of teledentistry

Participants demonstrated notably positive perceptions of teledentistry in all aspects (Table 2), with an overall mean rating of 4.33 out of 5 (SD = 0.61). In addition to its strength in preventing COVID-19 infection (Mean = 4.56, SD = 0.77), teledentistry was highly valued for its time saving and financial saving capabilities, receiving a score of 4.41 (SD = 0.84) and 4.37 (SD = 0.89), respectively. Among the different groups of stakeholders, orthodontic instructors indicated the highest perceived benefits, with an overall score of 4.56 (SD = 0.41), followed by residents (Mean = 4.29, SD = 0.62) and practitioners (Mean = 4.19, SD = 0.70).

**Table 2:** Self-perceived benefits of teledentistry

Self-perceived benefits	Instructors Mean (SD)	Practitioners Mean (SD)	Residents Mean (SD)	Total Mean (SD)
Decrease risk of COVID-19 infection	4.80 (0.56)	4.47 (0.64)	4.50 (0.88)	**4.56 (0.77)**
Time saving	4.67 (0.49)	4.13 (0.99)	4.42 (0.87)	**4.41 (0.84)**
Financial saving	4.73 (0.46)	4.06 (1.03)	4.31 (0.92)	**4.37 (0.89)**
Relief of patient concern	4.40 (0.74)	4.20 (0.68)	4.14 (0.90)	**4.21 (0.81)**
Close monitoring/follow-up	4.20 (0.77)	4.07 (0.80)	4.08 (0.77)	**4.11 (0.77)**
**Overall**	**4.56 (0.41)**	**4.19 (0.70)**	**4.29 (0.62)**	**4.33 (0.61)**

**Note:** Perceptions on a five-point scale, ranging from ‘Strongly disagree’ (1) to ‘Strongly agree’ (5)

#### Self-perceived concerns in the use of teledentistry

Participants rated concerns in the use of teleorthodontics, with variations across various aspects (Table 3). The efficacy of communication devices was perceived as the most significant concern, with a score of 4.14 (SD = 0.86). In contrast, participants expressed the least concern about missed appointments of their patients (Mean = 2.76, SD = 1.12). Among various stakeholder groups, orthodontic residents exhibited the highest self-perceived concerns, with an overall score of 3.78 (SD = 0.73), followed by practitioners (Mean = 3.57, SD = 0.64) and instructors (Mean = 3.44, SD = 0.54).

**Table 3:** Self-perceived concerns of teledentistry

Self-perceived concerns	Instructors Mean (SD)	Practitioners Mean (SD)	Residents Mean (SD)	Total Mean (SD)
Efficacy of communication devices	4.13 (1.06)	4.13 (0.64)	4.14 (0.87)	**4.14 (0.86)**
Diagnostic accuracy	3.73 (0.88)	3.87 (0.99)	4.08 (0.94)	**3.95 (0.94)**
Data privacy and confidentiality	3.80 (1.21)	3.67 (1.05)	3.72 (1.00)	**3.73 (1.05)**
Technical issues	3.13 (1.19)	3.80 (1.01)	3.89 (1.04)	**3.70 (1.10)**
Missed appointment	2.40 (0.99)	2.40 (0.99)	3.06 (1.17)	**2.76 (1.12)**
**Overall**	**3.44 (0.54)**	**3.57 (0.64)**	**3.78 (0.73)**	**3.66 (0.68)**

**Note:** Perceptions on a five-point scale, ranging from ‘Strongly concern’ (1) to ‘Strongly concern’ (5)

### The implementation of teledentistry training into orthodontic postgraduate curriculum

The topics that should be included in the curriculum were explored by using a check-box with multiple responses (Table 4). The findings indicated that the majority of participants considered ‘Ethical and legal concerns for teledentistry’ (83.33%), followed by ‘Orthodontic services in teledentistry’ (80.30%) and ‘Databases in teledentistry’ (78.79%).

**Table 4:** Topics to be included in teledentistry training

**Topics to be included in teledentistry trai**ning	**Instructors** **(n)**	**Practitioners** **(n)**	**Residents** **(n)**	**Total** **(n)**
Ethical and legal concerns for teledentistry	15	13	27	**55**
Orthodontic services in teledentistry	12	10	30	**53**
Databases in teledentistry	10	11	31	**52**
Platforms utilized in teledentistry	9	11	25	**45**
Tools and devices used for teledentistry	9	11	19	**39**
Teledentistry in dental education	5	8	18	**31**
Compositions of teledentistry system	6	9	12	**28**
Teledentistry during the COVID-19 pandemic	5	9	11	**25**
Historical evolution of teledentistry	2	5	2	**9**

### Qualitative phase

Following the framework analysis of qualitative data, there were four emerging themes, which were ‘Current applications of teledentistry in orthodontic practice’, ‘Advantages of teledentistry in orthodontic practice’, ‘Concerns regarding the use of teledentistry in orthodontic practice’, and ‘Suggestions for the design of teledentistry training in orthodontic education’.

## Current applications of teledentistry in orthodontic practice

Most participants conveyed that they had experiences with teledentistry in their orthodontic practice. This not only involved providing virtual consultations to their orthodontic patients but also allowed them to communicate online with other dental specialists.

### Dentist-dentist communication

Participants reported using teledentistry to communicate with dental professionals from various specialties, particularly for multidisciplinary cases that required comprehensive treatment plans in orthodontic care.

*“I frequently use teledentistry in consultations with other dental specialists, discussing matters like patient referrals or seeking input from surgeons for artificial eruption prior to surgical procedures. The consultations may extend to prosthodontists when considerations for dentures or implant placement are needed.” [Practitioner 3]*

### Dentist-patient communication

#### Initial examination and history taking

Participants reported they implemented teledentistry as an initial screening tool before commencing actual treatment. This approach enabled orthodontists to gather preliminary patient information, present possible alternatives for orthodontic treatment plans, and provide estimates of associated expenses. These tasks could be performed without the necessity for onsite dental appointments.

*“With virtual consultations, orthodontists can initially gather patient information and give some helpful advice about patient problems and orthodontic treatment plan, which the dentist and patient are not necessary to meet face-to-face.” [Practitioner 2]*

#### Emergency management

Teledentistry was perceived as a valuable approach to promptly and effectively manage orthodontic emergencies, thereby enhancing the accessibility and continuity of orthodontic care. In scenarios such as archwire impingement on the gums or bracket detachment, virtual consultations facilitated tailored guidance from orthodontic professionals. These consultations enabled patients to receive specific recommendations for addressing immediate concerns, mitigating discomfort, and implementing interim measures until an in-person appointment can be arranged.

*“When my patients feel discomfort due to the archwire poking the gingiva, I can suggest them via teleorthodontics to consider purchasing orthodontic wax from a nearby dental office to ease the irritation between appointments. This way, they can feel better before their next in-person visit.” [Instructor 1]*

#### Follow-up

Teledentistry emerged as a valuable tool for conducting follow-up visits throughout the course of orthodontic treatment, extending its utility to the monitoring of tooth movement and treatment progress. In cases of obstructive sleep apnea, teledentistry facilitates continuous monitoring, especially in situations where on-site appointments are impractical. This capability ensures ongoing assessment and management, thereby contributing to comprehensive care delivery in scenarios where in-person evaluations may be challenging.

*“With teledentistry, I could ensure that treatment progress aligns with expectations. Patients can conveniently share photos or videos, allowing orthodontists to provide remote guidance without the need for an in-person dental examination.” [Instructor 3]*

*“Teledentistry serves as a valuable tool for follow-up in obstructive sleep apnea cases, particularly during the COVID-19 pandemic, to ensure that patients can receive necessary support without the need for in-person visits.” [Resident 1]*

## Advantages of teledentistry in orthodontic practice

The potential advantages derived from the qualitative findings on the applications of teleorthodontics encompassed noteworthy benefits, including time and financial savings, reduced infection risks, and immediate emergency management.

### Time saving

The majority of participants perceived teledentistry as an approach to achieve time saving by reducing travel requirements and waiting times. Although patients may need to be placed on a waiting list for virtual consultations, they can effectively manage their time for other productive activities at their convenience, as opposed to the conventional practice.

*“Patients can now communicate with their dentists remotely, eliminating the need for unnecessary travel time and waiting in crowded waiting rooms.” [Resident 4]*

### Financial saving

Participants acknowledged the potential of teledentistry to alleviate financial burdens for stakeholders and to enhance accessibility to orthodontic treatment. This was particularly evident in the waiving of transportation costs and sterile equipment fees. It also helped mitigate income loss incurred by patients when taking leave from work.

*“Teledentistry not only reduces travel costs but also allows patients to maintain their careers without the loss of income.” [Instructor 2]*

### Minimizing infection risks

Teledentistry emerged as a valuable tool for maintaining dental procedures during the COVID-19 pandemic. It played a crucial role in mitigating the risk of coronavirus transmission linked to aerosol production within dental hospitals or clinics. Additionally, the reduction in transportation needed contributes to a reduced risk of exposure to COVID-19 or other infectious diseases.

*“Imagine a situation where a pregnant woman faces problems with her dental braces. Teledentistry becomes a practical solution, letting her deal with the issue from a distance without risking exposure to potential infections from going to a dental clinic.” [Practitioner 3]*

### Immediate relief from pain and worry for orthodontic emergencies

The alleviation of pain and worry through instant management in emergency orthodontic cases was reported as an additional factor that promoted the utilization of teledentistry. This platform facilitated prompt consultation and management when a patient needed immediate care for unexpected pain. Although there were instances where management might not be feasible at home, teledentistry primarily relieved concerns of those patients.

*“Teledentistry is really helpful in promptly addressing pain and worry in emergency orthodontic cases. It provides instant management and consultations when a patient needs immediate care for unexpected pain.” [Resident 2]*

## Concerns regarding the use of teledentistry in orthodontic practice

Despite the positive perceptions surrounding teledentistry, participants disclosed specific concerns and challenges associated with its adoption in orthodontics. These apprehensions highlighted the nuanced considerations that need to be addressed to ensure the successful integration of teledentistry within the orthodontic field.

### Diagnostic accuracy of teledentistry

In contrast to onsite orthodontic examinations, virtual consultations relied on a two-dimensional perspective, introducing inherent limitations that present challenges in capturing the full details observed in patients. This limitation had the potential to impact the decision-making process, as the absence of depth and the variability in angles could create difficulties for dentists in forming a comprehensive and precise assessment.

*“All patient details are shown in two-dimensions. … Sometimes when a patient takes a picture with the camera closer to themselves, their face may appear larger in the photo, resulting in a visual difference from the real person.” [Practitioner 1]*

*“The clarity of details from teleorthodontics cannot be compared to real patients. The quality of patient information from teledentistry is varied and could be inadequate, sometimes leaving me unsure about the provided diagnosis.” [Resident 2]*

*“For more complicated cases, such as craniofacial deformities, particularly for patients with cleft conditions … It is better to have the patient come in for a proper assessment.” [Instructor 1]*

### Confidentiality and privacy of virtual consultations

Confidentiality and privacy emerged as significant concerns among the majority of participants. The consensus among dental practitioners was that the teledentistry platform in orthodontics should prioritize patient privacy while also protecting the confidentiality of practitioners. Furthermore, there was a potential risk of patients recording and selectively sharing portions of virtual consultations, leading to potential misunderstandings. Thus, both provider and patient consent should be considered mandatory for each virtual consultation session to address these privacy considerations effectively.

*“We need to ensure that it is highly secure, so the patient data remain confidential, without any risk of leaks. This level of security is also vital to protect the dentist and their information.” [Instructor 1]*

*“I sometimes feel uncomfortable with virtual consultations, given the possibility that patients might record and share them publicly or on social media. [Resident 2]*

### Technological literacy of stakeholders

Participants expressed concern that the complexity of technology can indeed pose a significant challenge and limitation in the adoption of teleorthodontics. Individuals with limited access or familiarity with technology, especially the elderly, may face difficulties in managing teledentistry and might be hesitant to utilize it.

*“Some people including both practitioners and patients may not be familiar with or comfortable using the technology required for teleorthodontics” [Instructor 2]*

*“It can be challenging, especially for patients who have no experiences with teledentistry … younger patients may adapt easily, but teaching older patients can be quite difficult.” [Resident 1]*

### Awareness of teledentistry services

Participants emphasized variations in awareness and support for teledentistry services across different countries. There was a notable difference in awareness of teledentistry between developed and developing countries. They also suggested that the establishment of relevant regulations, dental protection agencies, and healthcare insurance providers was essential to foster the adoption and implementation of teledentistry, as observed in more advanced countries.

*“In Thailand, awareness of teledentistry is limited … dental protection and healthcare insurance as well as their regulations and frameworks should be considered for these services.” [Instructor 1]*

## Suggestions for the design of teledentistry training in orthodontic education

Participants highlighted the necessity of training before the implementation of teledentistry in orthodontic practice, emphasizing on enhancing patient management and workflow. The qualitative data further delved into the expected learning outcomes of training, delivery methods, and assessment strategies, providing valuable insights into the reasoning behind each perspective. This comprehensive approach aimed to address the diverse needs and challenges faced by practitioners, ensuring effective adoption of teledentistry.

### Expected learning outcomes (Knowledge and skills required for teleorthodontics)

Participants emphasized several essential learning outcomes to supporting orthodontic practitioners in effectively applying teledentistry in their practice. The discussion initially centered around fundamental elements necessary for the successful integration and utilization of teledentistry within orthodontic practice. These encompassed the compositions of teledentistry system, potential platforms, system operation, and considerations related to data storage.

*“The topics I could consider to include are the introduction of what teledentistry is and its composition, the potential platforms for teledentistry, system operation, and data storage and management.” [Resident 2]*

*“Having training for teledentistry is beneficial, and I am eager to receive the training as well. Older generations, like myself, may face challenges in adapting to new technologies.” [Practitioner 1]*

*“It is important to understand the workflow before using teledentistry with the patient so that the orthodontist can work more efficiently.” [Resident 4]*

Additional topics recommended for inclusion in teledentistry training were laws and regulations to ensure the security and privacy of patient data, procedures for obtaining necessary consent, and considerations related to dental ethics. Participants also suggested to include effective communication emphasizing appropriate manners, active listening, and the maintenance of a proper doctor-patient relationship when using teledentistry. These considerations contributed to a comprehensive and well-rounded training program for practitioners engaging in teledentistry.

*“I believe training is necessary … because orthodontists can have access to patients' personal information, they should know how to manage such situations in accordance with dental ethics.” [Resident 1]*

*“Laws and communication … I believe that effective communication is necessary because, as humans, not robots, both practitioners and patients are bound by human factors such as empathy and understanding.” [Instructor 1]*

### Delivery techniques

Participants suggested that the delivery techniques for teledentistry training could involve a combination of online and onsite lectures, complemented by simulation-based learning before applying the system with actual patients. There was also mention of the possibility of establishing a separate training course dedicated to teledentistry. Conversely, some participants proposed integrating the teledentistry training into an existing subject within the framework of an established curriculum. The varied perspectives highlighted the need for adaptable and comprehensive training approaches to meet the diverse preferences and needs of practitioners.

*“The introduction, laws, and regulations could be covered in a lecture format, whereas the platform usage could be addressed through hands-on training. The communication aspect, being a soft skill that varies among individuals, may not necessarily require formal training.” [Resident 2]*

*“Role-plays can be effective in training us to interact with simulated patients via teledentistry, helping us practice and refine our communication skills in various scenarios.” [Resident 4]*

*“The incorporation of teledentistry into a dental school could provide residents with valuable experience in virtual consultations. Having a checklist for students to follow, along with observation and feedback from instructors, would further enhance their learning.” [Instructor 3]*

*“Short courses outside dental schools can provide opportunities for both residents and practitioners to receive training and share experiences in teledentistry.” [Practitioner 2]*

### Assessment strategies

Participants discussed various assessment strategies to ensure that learners could achieve expected learning outcomes. Written examinations were considered suitable for evaluating the cognitive domain after lectures. Report writing emerged as a valuable means to assess comprehension and application, especially regarding the establishment of a teledentistry system in orthodontic practice. Recognizing that virtual consultations involved the affective domain, particularly in communication skills, options such as observation during real-world activities were suggested. Additionally, the design of simulated scenarios was proposed to provide a structured framework for learners to showcase their skills, enabling observation and evaluation by experts.

*“Written examinations can figure out if learners are really getting what they have learnt from the class. Like, are they remembering and actually using the stuff they learned?” [Resident 4]*

*“To assess the readiness of learners, a simulated scenario of teleorthodontic practice can be created. They can then complete this scenario to evaluate their preparedness and proficiency in the context of teledentistry.” [Instructor 3]*

*“Observation by experts during simulation-based learning and real-world activities where students can demonstrate their performance to navigate virtual consultations effectively, demonstrating their communication skills.” [Instructor 2]*

### Conceptual framework for implementing teledentistry training in orthodontic education

**Fig. 1:**
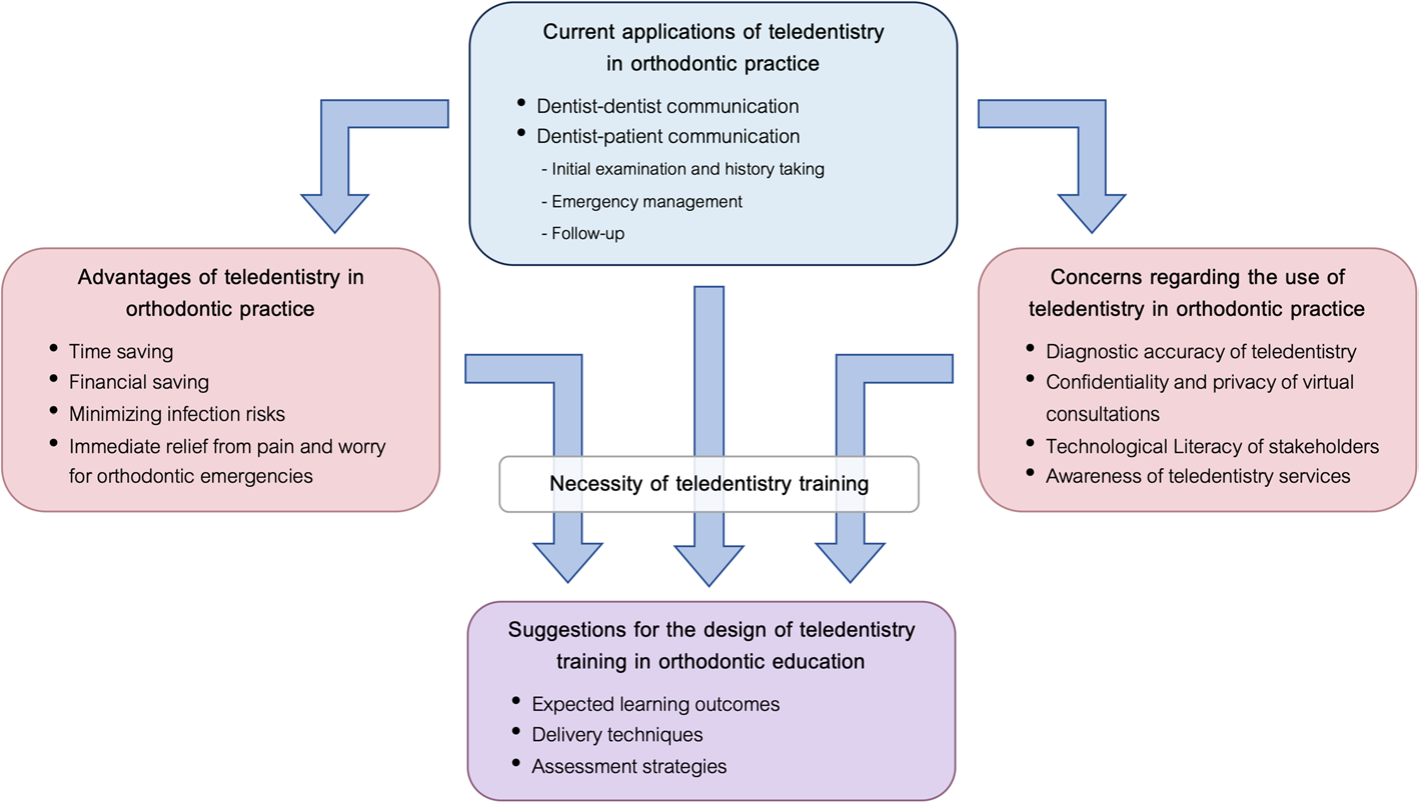
Conceptual framework for implementing teledentistry training in orthodontic education

A conceptual framework was systematically developed through the analysis of qualitative data (Fig. 1). This framework illustrates the relationships between the identified thematic elements and highlights how these elements inform the integration of teledentistry training into postgraduate orthodontic curricula. The framework is grounded in the concept of outcome-based education, suggesting how teledentistry training can be integrated based on its applications in clinical practice. The applications of teledentistry in orthodontic practice, along with its advantages and concerns, inform the expected learning outcomes. The training should emphasize the importance of learners actively constructing their competencies through experiencing and reflecting on teledentistry training which includes lectures, practical experiences in simulated situations, and clinical practice.

The conceptual framework comprehensively outlines the current applications, advantages, concerns, necessity, and suggestions for teledentistry training in orthodontic practice. It highlights how teledentistry facilitates efficient doctor-doctor and doctor-patient communication, saving time and costs while minimizing infection risks, particularly pertinent during situations like the COVID-19 pandemic. However, concerns such as diagnostic accuracy, confidentiality, technological literacy, and awareness of services need careful consideration. The necessity for teledentistry training is highlighted to ensure practitioners are equipped with skills to navigate these challenges effectively. Suggestions for training design emphasize clear learning outcomes and a diverse instructional approach combining didactic teaching, simulations, and virtual consultations in real situations to optimize learning experiences. Overall, while teledentistry offers significant benefits, addressing associated concerns through comprehensive training is crucial for its successful integration into orthodontic education and practice.

## Discussion

All the stakeholders involved in this research expressed positive perceptions regarding the implementation of teledentistry in orthodontic practice where several advantages of teleorthodontics were highlighted primarily centered around time saving, financial savings, reduced infection risks, and instant management for emergency cases. This trend aligns with findings from previous research spanning approximately a decade [4, 18–22]. Teledentistry can also play an important role for dentist-dentist communication, particularly in supporting treatment planning for complex cases and facilitating consultations between orthodontists and other dental specialists [6]. This led to an increasing use of teledentistry during the past five years, particularly in orthodontic practice [5]. This trend holds the potential to enhance patient satisfaction by maximizing the benefits derived from teledentistry throughout the course of orthodontic treatment.

This research revealed that the participants had realized key concerns in teleorthodontics. Technological literacy, particularly among new users, presents challenges due to the elevated level of technological complexity, leading to delays in system initiation [4, 12]. Confidentiality has emerged as a major concern, primarily due to the potential risks associated with online treatment session recordings by patients without consent and a concurrent lack of awareness regarding patient privacy and security during virtual consultations [12]. This underscores the need for enhanced privacy awareness in the context of teledentistry implementation. Participants also noted the use of non-compliant social media platforms, lacking adherence to HIPAA regulations and compromising patient information security [8]. These suggestions highlight the need for a dedicated platform or official account to ensure organized and credible teledentistry services in orthodontic practice, emphasizing the importance of addressing technological and ethical aspects. In addition, standardizing teledentistry services in orthodontics becomes crucial to address the current lack of consistency in practices, potentially impacting patient care and outcomes.

Incorporating teledentistry training into postgraduate orthodontic education is increasingly necessary, given its potential to enhance patient management, streamline workflow, standardize services, and improve access to orthodontic care while upholding ethical standards. The needs for dentists to undergo specific teledentistry training is emphasized by participants, aligning with the American Dental Association Principles of Ethics and Code of Professional Conduct [23]. Therefore, up-coming generations of orthodontic practitioners should be equipped with essential competencies to achieve full potential of teledentistry [20, 24]. However, the evidence retrieved from this research demonstrated the current limitations in teledentistry training, together with the insufficient knowledge and awareness among dental practitioners regarding teledentistry [12, 25]. Consequently, this research emphasizes the urgency of integrating standardized training into postgraduate orthodontic education to prepare future orthodontists for the evolving 21st-century landscape.

In designing teledentistry training, the initial step involves establishing clear and expected learning outcomes to guide the design of the course. The principal distinction between onsite and virtual consultations lies in the communication pathway. Participants emphasized the significance of topics such as communication with appropriate manners, active listening, and maintaining a strong doctor-patient relationship. Effective communication is critical for accurate diagnosis and the formulation of suitable treatment plans [26]. The doctor-patient relationship is also essential for delivering quality healthcare [27]. Moreover, participants underscored the importance of addressing ethics, laws, and the establishment of a teledentistry database. Existing evidence supports the awareness among dental professionals of the need to comprehend legal and ethical aspects and ensure the accurate documentation of patient data [28]. The suggested training topics for teleorthodontics encompass the composition of the teledentistry system, potential platforms for orthodontic teledentistry, system operation, data storage, legal regulations ensuring the security and privacy of patient data, required consent procedures, and considerations of dental ethics. These comprehensive topics provide a robust foundation for teledentistry training, preparing practitioners to effectively address the ethical, legal, and technical challenges of this evolving field.

To facilitate learners in achieving the expected learning outcomes, participants suggested a combination of training delivery and assessment strategies. These strategies encompass a blend of online and onsite lectures, supplemented by hands-on simulations conducted before the application of the system in clinical practice [12, 25, 29]. Simulations could offer learners the opportunity to gain practical experiences within safe learning environments [30]. Within these simulated situations, gaming features can be incorporated to enhance motivation and engagement, where immediate feedback mechanisms enable learners to iteratively improve their skills from their failures [31–33]. These simulated scenarios not only provide a standardized environment for learners to demonstrate their competence in teledentistry but also enable observation and evaluation by experts [34]. This combination of training and assessment strategies ensures that learners attain competence before employing teledentistry with orthodontic patients.

While this study employed a sequential explanatory mixed methods design with a focus on prioritizing validity, reliability, and transparency to ensure the robustness of data collected from relevant stakeholders, it is essential to acknowledge and discuss a few unavoidable limitations that may have influenced the research quality. Notably, this research was conducted with only participants from the Orthodontic Clinic at Mahidol University. Therefore, it is recommended that further research should be undertaken across multiple sites with a larger sample size to standardize learning outcomes, providing a more comprehensive guideline for designing a teledentistry training course. Additionally, the conceptual framework developed from the qualitative data in this research should be further validated through quantitative research to construct a statistical prediction model. Furthermore, learning interventions for teledentistry training ought to be designed and evaluated to confirm their effectiveness in dental education.

## Conclusions

This research supports the implementation of teledentistry in orthodontic practice. The findings indicate that participants had awareness of both advantages and concerns associated with the use of teledentistry. Additionally, the research reveals the current limitations of teledentistry in orthodontic education. The conceptual framework developed in this research highlights the need for teledentistry training in orthodontic curricula. It also emphasizes the importance of expected learning outcomes, alongside a combination of training delivery and assessment strategies, to adequately prepare learners for the use of teledentistry in orthodontic practice. However, further research is necessary to establish standardized guidelines for teledentistry education.

## Data Availability

The data that support the findings of this study are available from the corresponding author, up-on reasonable request. The data are not publicly available due to information that could compromise the privacy of research participants.
